# Clustering patterns of physical activity, sedentary and dietary behavior among European adolescents: The HELENA study

**DOI:** 10.1186/1471-2458-11-328

**Published:** 2011-05-17

**Authors:** Charlene Ottevaere, Inge Huybrechts, Jasmin Benser, Ilse De Bourdeaudhuij, Magdalena Cuenca-Garcia, Jean Dallongeville, Maria Zaccaria, Frederic Gottrand, Mathilde Kersting, Juan P Rey-López, Yannis Manios, Dénes Molnár, Luis A Moreno, Emmanouel Smpokos, Kurt Widhalm, Stefaan De Henauw

**Affiliations:** 1 Department of Public Health, Ghent University, Ghent, Belgium; 2 Institut für Ernährungs- und Lebensmittelwissenschaften - Ernährungphysiologie, Rheinische Friedrich Wilhelms Universität, Bonn, Germany; 3 Department of Movement and Sport Sciences, Ghent University, Ghent, Belgium; 4 Department of Physiology, School of Medicine, University of Granada, Granada, Spain; 5 Institut Pasteur de Lille, Lille, France; 6 National Research Institute on Food and Nutrition, Rome, Italy; 7 Inserm U995, IFR114, Faculty of medicine, Université de Lille2, Lille, France; 8 Research Institute of Child Nutrition Dortmund, Rheinische Friedrich-Wilhelms-Universität Bonn, Bonn, Germany; 9 Growth, Exercise, Nutrition and Development (GENUD) research Group, E.U., Ciencias de la Salud, Universidad de Zaragoza, Zaragoza, Spain; 10 Harokopio University, Athens, Greece; 11 Pécsi Tudemányegyetem, Pecs, Hungary; 12 University of Crete School of medicine, Crete, Greece; 13 Medical University of Vienna, Vienna, Austria

## Abstract

**Background:**

Evidence suggests possible synergetic effects of multiple lifestyle behaviors on health risks like obesity and other health outcomes. A better insight in the clustering of those behaviors, could help to identify groups who are at risk in developing chronic diseases. This study examines the prevalence and clustering of physical activity, sedentary and dietary patterns among European adolescents and investigates if the identified clusters could be characterized by socio-demographic factors.

**Methods:**

The study comprised a total of 2084 adolescents (45.6% male), from eight European cities participating in the HELENA (Healthy Lifestyle in Europe by Nutrition in Adolescence) study. Physical activity and sedentary behavior were measured using self-reported questionnaires and diet quality was assessed based on dietary recall. Based on the results of those three indices, cluster analyses were performed. To identify gender differences and associations with socio-demographic variables, chi-square tests were executed.

**Results:**

Five stable and meaningful clusters were found. Only 18% of the adolescents showed healthy and 21% unhealthy scores on all three included indices. Males were highly presented in the cluster with high levels of moderate to vigorous physical activity (MVPA) and low quality diets. The clusters with low levels of MVPA and high quality diets comprised more female adolescents. Adolescents with low educated parents had diets of lower quality and spent more time in sedentary activities. In addition, the clusters with high levels of MVPA comprised more adolescents of the younger age category.

**Conclusion:**

In order to develop effective primary prevention strategies, it would be important to consider multiple health indices when identifying high risk groups.

## Background

Unhealthy dietary habits and a lack of physical activity (PA) are well known risk factors for several chronic diseases (e.g. cardiovascular diseases, type 2 diabetes) [1,2]. Together with the adoption of sedentary behaviors, they are likely major contributors to the increasing prevalence of obesity in youth [3]. Television watching or screen time, mostly used as a measure of sedentarism, have also been related to the consumption of foods advertised on television and the intake of energy-dense foods [4]. Furthermore, those health behaviors have the tendency of tracking from childhood into adulthood [5,6]. Children and adolescents should accumulate at least 60 minutes of moderate to vigorous PA (MVPA) per day [7]. According to previous research no more than one third of the adolescents seemed to achieve this PA recommendations [8,9]. Also, a decline in PA from childhood to adolescence has previously been shown [10,11]. Moreover, the American Academy of Paediatrics has recommended that children spend no longer than two hours per day on sedentary activities [12]. From the HBSC (Health Behavioural in School-aged Children) study and recent findings from the HELENA (Healthy Lifestyle in Europe by Nutrition in Adolescence) study (unpublished data), it was shown that a high proportion of the adolescents did not meet this recommendation [9]. The HBSC Study also revealed that food habits of adolescents are characterized by a high consumption of sweets and soft drinks, breakfast skipping, and a low consumption of fruits and vegetables [9]. These results indicate the need for a systematic promotion of healthy lifestyles starting from childhood. However, in order to launch effective promotion strategies, it is important to identify the needs and population groups at risk first. Therefore, it is necessary to gain insight on the dominant socio-demographic correlates for PA, sedentary behavior and adverse dietary patterns.

Furthermore, little is known about the co-occurrence (clustering) of those behaviors and the association with socio-demographic factors because most researchers had mostly focused separately on the prevalence of each one of these health-related behaviors and their associated demographic characteristics. Another important issue is a possible synergetic effect of the co-occurrence of multiple health behaviors on the risk of chronic diseases and other health outcomes. Therefore, a better insight in the clustering of multiple health behaviors, could help to identify groups who are at particular risk in developing chronic diseases. Since adolescence is a critical period during lifetime in adopting health behaviors and also because those behaviors may track into adulthood, the study of multiple health indices should be a public health priority.

The aim of the present study is to examine the prevalence and clustering of PA, sedentary and dietary behavior among European adolescents. In addition, to investigate if the identified clusters could be characterized by socio-demographic factors like age, gender, Body Mass Index (BMI), parents' education level, and Familial Affluence Scale (FAS) score.

## Methods

### Study design

Data was derived from the HELENA-Cross Sectional Study (CSS), which was conducted in 10 European cities (Athens in Greece, Dortmund in Germany, Ghent in Belgium, Heraklion in Crete, Lille in France, Pecs in Hungary, Rome in Italy, Stockholm in Sweden, Vienna in Austria, and Zaragoza in Spain) from 2006 to 2007. The main objective of the HELENA-CSS study was to obtain reliable and comparable data of a large sample of European adolescents on a variety of nutrition and health related parameters on a standardized procedure [13].

The study was approved by the Ethical Committee of each city involved [14]. A signed informed consent was obtained from both the adolescents and their parents. Details on sampling procedures and study design of the HELENA study have been reported elsewhere [13,15].

### Measurements

#### Socioeconomic status (SES)

The FAS index was used as an indicator of the adolescents' material affluence and a predictor of their health outcomes. The FAS index was slightly adapted to the HELENA population. Family car ownership, having an own bedroom, internet availability, and computer ownership are variables included in the FAS. The scale has been shown to be a valid indicator for the socioeconomic and material circumstances of adolescents [16]. A score from 0-3 reflects low familial wealth, 4-5 medium and 6-8 high familial wealth.

The adolescents reported their parents' educational level as primary education, lower secondary education, higher secondary education or higher education/university degree. This 4-point scale was recoded into a 2-point scale, namely a low (lower education and lower secondary) and high (higher secondary and higher education/university degree) score.

#### International Physical Activity Questionnaire for Adolescents (IPAQ-A)

An adapted version of the International Physical Activity Questionnaire (IPAQ), was used to assess PA of the last 7 days, namely the IPAQ-A. The original IPAQ is a self-administered questionnaire and was originally developed as a cross-national monitoring of PA and inactivity in adults (15-69 years). The questionnaire is a valid and reliable tool to measure PA in a European adult population [17]. To adapt the questionnaire to the HELENA population, questions about PA at work were replaced by questions about PA at school. Furthermore, the module about activities in the household domain had been shortened. Only one question (versus three in the original IPAQ) about PA in the garden or at home remained. Also, the order of PA intensities was changed, to avoid over reporting [17]. The time spent at walking was asked before the time spent at vigorous and moderate PA intensity (versus vigorous, moderate and walking in the original IPAQ). Total minutes per week were computed for MVPA based on the guidelines for data processing and analyses of the IPAQ (http://www.ipaq.ki.se/ipaq.htm). Data were cleaned and truncated based on previous research [18]. The IPAQ-A was tested for validity by Hagströmer et al. [19] by comparing the IPAQ-A results with accelerometer data. Significant, but modest correlations (± 0.20) were found and a higher validity in the older adolescents in comparison with the younger ones was revealed.

#### Sedentary questionnaire

Sedentarism was assessed by a self-reported HELENA questionnaire. The questionnaire included daily minutes of the following sedentary items: television viewing, playing with computer games, playing with console games, use of internet for non-study reasons, use of internet for study, and studying/homework (lessons not included). The average time spent per day in those sedentary activities was calculated. The sedentary questionnaire was tested for reliability and it was shown that the questionnaire is a reliable tool to be used in adolescents [20]. Furthermore, the questionnaire also allowed correctly classifying daily inactive time in boys, when compared with accelerometry (unpublished data).

#### HELENA-Dietary Assessment Tool (HELENA-DIAT)

A self-administered computerized 24-h recall, named HELENA-DIAT, was used to obtain dietary intake data. This 24-h dietary recall was completed during school time and was assisted by dieticians/researchers, who instructed the participants on how to fill in this recall as accurate as possible. The adolescents were allowed to ask questions and assistance and after completion, the recall was checked for completeness and correctness. Every participant was asked to fill in the HELENA-DIAT twice in a time-span of 2 weeks. A validation study by Vereecken et al. [21] indicated that the YANA-C, a former version of the HELENA-DIAT, showed highly significant correlations (Rs = 0.86-0.91) for all nutrients and energy intake with an interviewer-administered HELENA-DIAT interview. Furthermore, agreement in categorizing the respondents as consumers and non-consumers for the foods was found to be high (kappa statistics ≥ 0.73). The HELENA-DIAT tool has been indicated as a good method to collect detailed dietary information from adolescents and was received well by the study participants [22]. In addition, the European Consumption Survey Method (EFCOSUM) project selected a repeated 24-h recall as the most suitable method to get population means and distributions [23].

Based on the data derived from the HELENA-DIAT, a Diet Quality Index for Adolescents (DQI-A) was calculated. The basic principles of a good and healthy diet are considered in the DQI-A, namely "dietary equilibrium" (adequacy and moderation), "dietary diversity", "dietary quality" and a "meal index". The dietary equilibrium expresses a balance in food intake and a diet is in balance when an adequate but moderate intake of all food groups is reassured. Dietary diversity expresses the degree of variation in the diet. Furthermore, the choice of optimal food quality is expressed in the dietary quality. Finally, the meal index reflects the frequency of consumption of meals (breakfast, lunch and dinner). To calculate the DQI-A, the scores of those four categories were summed and divided by four, resulting in a score ranging from -25 to 100%. The higher the score, the higher the adolescents' quality of dietary habits. The calculation of the DQI-A was based on a previously developed DQI for preschoolers by Huybrechts et al. [24]. Those researchers also indicated a reasonable validity and reproducibility of the DQI score and concluded that it can be seen as a good estimate for the dietary quality of the study population.

#### Anthropometric measurements

Weight and height of the adolescents were measured by trained researchers in a standardized way [25]. Weight was recorded to the nearest 0.1 kg, using an electronic scale (Type SECA 861) and height to the nearest 0.1 cm, using a telescopic height measuring instrument (Type SECA 225). Light indoor clothing could be worn, excluding shoes, long trousers and sweaters. BMI of the adolescents was calculated from their measured height and weight (BMI = weight divided by height squared, [kg/m²]). International age- and gender-specific cut points [26,27] were used to assess their BMI-category namely underweight, normal weight, overweight or obese.

### Participants

In the HELENA study, 3528 adolescents were recruited. Only those adolescents who completed at least 75% of the IPAQ-A and sedentary questionnaire and filled in the HELENA-DIAT for at least two days were included in the present study. In total, 1113 participants did not meet those inclusion criteria. Furthermore, Crete could not be included in the 24-h recall analyses since only a minority of the study population completed two 24-h recall days. Also Hungary was excluded from the 24-h recall analyses because no nutrient information was available and thus the standardized data cleaning procedures could be performed. Therefore, only 8 study centers could be included for the 24-h dietary recall analyses (Stockholm, Dortmund, Ghent, Lille, Athens, Rome and Zaragoza) and finally, 2084 cases (45.6% male) remained eligible for further analyses. The younger age category (12.5 - 14.99 years of age) consisted of 545 males and 648 females with a mean age of 13.8 (±0.7) years. The older age group (15 - 17.5 years of age) consisted of 406 males and 485 females with a mean age of 16.0 (±0.7) and 15.9 (±0.6) years respectively. The excluded group of adolescents was equally distributed for sex (50.7% male). A higher percentage of adolescents with high educated parents was seen in the included compared to the excluded sample (65.2% (mother) and 60.7% (father) versus 55.2% (mother) and 50.2 (father) respectively). Also, the FAS was higher in the included adolescents (Low = 21.9%; Medium = 42.9%; High = 35.2%) compared to the excluded ones (Low = 37.0%; Medium = 38.1%; High = 21.2%). No differences were found between the included and excluded group for age (14.7 years (SD 1.20) and 14.7 years (SD 1.24) respectively), and BMI (21.2 (SD 3.65) and 21.7 (SD 3.83) respectively).

## Data analyses

Statistics were performed using SPSS for Windows (version 15.0 SPSS Inc., Chicago, IL, USA). To identify clusters with similar dietary, PA and sedentary habits, a combination of hierarchical and non-hierarchical clustering analysis was used [28]. Because the three health indices had different arithmetic scales, z-scores of all indices were calculated to standardize the data set before clustering. In a first step, hierarchical cluster analysis was carried out using Ward's method, based on squared Euclidian distances. This step was used to identify and compare several possible cluster solutions and to provide information necessary for the following procedure, a non-hierarchical *k*-means clustering procedure. This second step is used to further fine-tune the preliminary cluster solution, obtained by the hierarchical clustering. A third step was the examination of the stability of the final cluster solutions. This was done by randomly dividing the total sample in two subsamples. The first two steps were then applied on both halves. Finally a Kappa degree of the cluster solutions of both subsamples with those of the total sample was calculated, to check for agreement.

Based on the three health indices, a five-cluster solution seemed the most adequate and stable representation of the study population. The Kappa statistics showed excellent agreement, к = 0.95 for one subsample and к = 0.92 for the second subsample. An ANOVA test, and a post hoc Bonferroni test, was used to investigate the differences between each cluster on all health indices.

Chi-square tests were used to examine associations between the identified clusters and gender. After stratifying the study population for gender, chi-square tests were also performed for the factors age, BMI, parents' education and FAS score.

## Results

**Figure 1:**
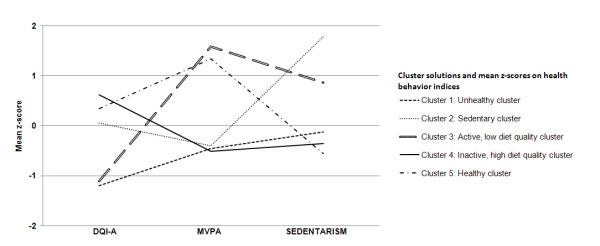
**Cluster solutions and mean z-scores on health behavior indices**.

Figure 1 represents the five defined clusters: (1) Unhealthy cluster (2) Sedentary cluster, (3) Active, low diet quality cluster, (4) Inactive, high diet quality cluster, and (5) Healthy cluster. The differences between the means of each cluster solution, reported in z-scores and row values (mean ± standard deviation), can be seen in table 1.

**Table 1:** Mean values of final cluster solutions and results of ANOVA and Bonferroni post-hoc test

	Cluster 1	Cluster 2	Cluster 3	Cluster 4	Cluster 5	F
**N(%)**	**430 (21)**	**247 (12)**	**152 (7)**	**877 (42)**	**378 (18)**	

**MVPA**	-0.46^b,d^	-0.40^e,g^	1.58^b,e,h,i^	-0.51^h,j^	1.34^d,g,i,j^	1098.69*
X ± SD (min/week)	452 ± 310	484 ± 369	1606 ± 410	422 ± 251	1467 ± 395	
**Sedentarism**	-0.12^a,b,c,d^	1.78^a,e,f,g^	0.86^b,e,h,i^	-0.36^c,f,h,j^	-0.55^d,g,i,j^	606.63*
X ± SD (min/day)	335 ± 137	708 ± 192	527 ± 176	288 ± 110	250 ± 113	
**DQI-A**	-1.20^a,c,d^	0.05^a,e,f,g^	-1.11^e,h,i^	0.62^c,f,h,j^	0.34^d,g,i,j^	694.46*
X ± SD (%)	46.4 ± 9.9	63.6 ± 11.1	47.7 ± 12.3	71.6 ± 7.1	67.6 ± 9.0	

MVPA = Moderate to Vigorous Physical Activity

DQI-A = Diet Quality Index for Adolescents

* P < 0.001

Cluster 1: Unhealthy cluster

Cluster 2: Sedentary cluster

Cluster 3: Active, low diet quality cluster

Cluster 4: Inactive, high diet quality cluster

Cluster 5:Healthy cluster

^a ^Significant difference between 1^st ^and 2^nd ^cluster

^b ^Significant difference between 1^st ^and 3^rd ^cluster

^c ^Significant difference between 1^st ^and 4^th ^cluster

^d ^Significant difference between 1^st ^and 5^th ^cluster

^e ^Significant difference between 2^nd ^and 3^rd ^cluster

^f ^Significant difference between 2^nd ^and 4^th ^cluster

^g ^Significant difference between 2^nd ^and 5^th ^cluster

^h ^Significant difference between 3^rd ^and 4^th ^cluster

^i ^Significant difference between 3^rd ^and 5^th ^cluster

^j ^Significant difference between 4^th ^and 5^th ^cluster

Adolescents in cluster 1 (Unhealthy cluster), cluster 2 (Sedentary cluster) and cluster 4 (Inactive, high diet quality cluster) spent the least amount of time in MVPA. A big difference between the 3 clusters is the time spent in sedentary activities. Adolescents from cluster 1 and 4 reported to spent less time than average in sedentary activities, while participants in cluster 2 indicated the highest sedentary level compared to all other cluster. Also for the DQI-A, all three clusters differed significantly. Adolescents in cluster 4 reported to eat the healthiest diets, while their peers in cluster 1 showed the lowest scores for the DQI-A. Adolescents in cluster 2 showed average scores for the DQI-A. Cluster 3 (Active, low diet quality cluster) can, together with cluster 1, be characterized by the lowest scores for the DQI-A. While adolescents from cluster 1 also showed the lowest MVPA levels, those in cluster 3 reported the highest time spent in MVPA. Participants in cluster 2 scored high for the sedentary index. Males and females in cluster 5 (Healthy cluster) reported to spend the lowest time in sedentary activities. They also showed the second best scores for the other two health indices. Participants in cluster 5 spent a high amount of time in MVPA, although significantly less than those in cluster 3. The group of adolescents in cluster 5 also reported to eat high quality diets, but their scores were significantly lower than those in cluster 4.

In table 2, the associations between the five clusters and the socio-demographic variables (gender, BMI, education level mother, education level father, FAS, and age category) are presented. For gender, significant chi-squares could be found. More males were represented in the Active, low diet quality cluster (cluster 3). The opposite cluster, namely the Inactive, high diet quality cluster (cluster 4) comprised more females. For the other three clusters more or less equal distributions could be found for male and female adolescents.

**Table 2:** Percentage of adolescents in each cluster for socio-demographic factors

		1	2	3	4	5	χ²
	**Gender (N)**						68.59*
**Total**	- Males (951)	49.5	51.0	63.8	35.8	53.2	
	- Females (1133)	50.5	49.0	36.2	64.2	46.8	

	**BMI (N)**						9.09
	- Underweight (50)	3.8	7.1	7.2	6.4	3.0	
	- Normal weight (670)	73.2	65.1	68.0	69.7	73.1	
	- Overweight (166)	17.4	17.5	18.5	17.5	17.4	
	- Obese (65)	5.6	10.3	7.2	6.4	6.5	
**Male**	**Education level mother (N)**						46.23*
	- Low (298)	44.1	39.0	50.6	23.7	23.2	
	- High (611)	55.9	61.0	49.4	76.3	76.8	
	**Education level father (N)**						25.02*
	- Low (293)	41.0	34.8	47.3	24.3	30.4	
	- High (596)	59.0	65.2	52.7	75.7	69.6	
	**FAS (N)**						13.23
	- Low (191)	21.6	22.2	20.6	19.7	17.4	
	- Medium (412)	43.7	42.1	29.9	43.3	50.2	
	- High (348)	34.7	35.7	49.5	36.9	32.3	
	**Age category (N)**						30.39*
	- Younger (12.5-14.99 yrs) (545)	44.6	57.9	62.9	55.4	70.6	
	- Older (15.0-17.5 yrs) (406)	55.4	42.1	37.1	44.6	19.4	

	**BMI (N)**						10.23
	- Underweight (91)	8.8	6.6	9.1	8.9	5.1	
	- Normal weight (834)	73.7	74.4	70.9	71.8	79.8	
	- Overweight (168)	16.1	15.7	16.4	14.7	12.4	
	- Obese (40)	1.4	3.3	3.6	4.6	2.8	
**Female**	**Educaction level mother (N)**						41.18*
	- Low (337)	44.7	40.2	47.1	24.8	24.6	
	- High (748)	55.3	59.8	52.9	75.2	75.4	
	**Educaction level father (N)**						29.19*
	- Low (368)	43.9	50.0	44.9	32.0	24.5	
	- High (668)	56.1	50.0	55.1	68.0	75.5	
	**FAS (N)**						10.33
	- Low (266)	27.6	24.8	18.2	21.7	24.9	
	- Medium (481)	42.9	46.3	41.8	43.7	35.6	
	- High (386)	29.5	28.9	40.0	34.6	39.5	
	**Age category (N)**						21.00*
	- Younger (12.5-14.99 yrs) (648)	50.7	56.2	74.5	54.7	68.4	
	- Older (15.0-17.5 yrs) (485)	49.3	43.8	25.5	45.3	31.6	

BMI = Body Mass Index FAS = Familial Affluence Scale

*P < 0.01

Cluster 1: Unhealthy cluster

Cluster 2: Sedentary cluster

Cluster 3: Active, low diet quality cluster

Cluster 4: Inactive, high diet quality cluster

Cluster 5: Healthy cluster

After stratifying the total sample by gender, significant chi-squares could be found for the education level of the mother and the father and for age category. For the parental education level the same trend can be seen in the male and female adolescents. The biggest difference in percentages between a low and high education level could be seen in the clusters with the high quality diets and lowest levels for sedentarism (cluster 4 and 5), irrespective of the time spent in MVPA. Adolescents reporting a low parental education level, were clearly less represented in cluster 4 and 5. This difference in distribution seemed to be stronger when looking at the education level of the mother. From the results it can also be seen that the Sedentary cluster (cluster 2), comprised more adolescents with high educated parents. This trend is especially true for the male group.

When splitting the study population for age, in both genders, the biggest differences were seen in the Active, low diet quality cluster and the Healthy cluster (cluster 3 and 5), which clearly represented more adolescents of the younger age category. Although less explicit, the Unhealthy cluster (cluster 1) comprised more older males. For BMI and FAS score no significant differences could be found in percentages between the five identified clusters.

## Discussion

The Healthy cluster (including 18% of the adolescents) was the only cluster, showing advisable scores for all three health indices. On the other hand, 21% of the adolescents had unfavorable scores for these lifestyles and are therefore represented in the Unhealthy cluster. These results revealed that several healthy lifestyle factors, which can be related to the prevalence of some chronic diseases and obesity [1-3], do not occur simultaneously among adolescents. Others have also indicated that many adolescents fail in meeting multiple recommended healthy lifestyle guidelines at once [29,30]. Pronk et al. showed that only 14.5% of the adolescents met the recommended guidelines for smoking, being physically active, consuming high-quality diet foods and having a healthy weight. In the study of Pearson et al. [30], only 6% of the adolescents achieved the recommendations for being physically active and for a daily consumption of fruits and vegetables and breakfast. Furthermore, the present finding of five meaningful clusters based on three health indices, may be an explanation for the discrepancies found in the literature examining the correlations between multiple health behaviors [31]. While some studies suggest that more active individuals are motivated to eat healthier [32], it could be that some people try to compensate, consciously or unconsciously via the complex neurological pathway of energy homeostasis [33], an unhealthy behavior by showing healthy habits for another dimension. The finding of two opposite clusters, namely the Active, low diet quality cluster and the Inactive, high diet quality cluster (together represents 49% of the adolescents), could support this hypothesis. It has also been suggested in literature that sedentary behavior replaces PA or vice versa. However, the high levels of MVPA and sedentarism in the Active, low diet quality cluster and the low levels for both indices in the Inactive, high diet quality cluster and the Unhealthy cluster show that there are no systematic negative association between time spent in MVPA and sedentary activities. This finding supports the suggestion that an association between PA and sedentarism does not automatically exist [34].

In addition, gender differences between the five clusters were found. Males were mainly represented in the cluster with the highest MVPA levels and the lowest diet quality scores, while the opposite was seen within the females. These gender differences are in line with those found in a cluster analyses by Sabbe et al. [35] in children 10 years of age. On top of the findings in the previous mentioned study, the present results also determine that females spent less time in sedentary activities compared to males. Although, previous research revealed that males and females showed different sedentary patterns [36]. Males spent more time watching TV and playing computer and console games, while females spent more time in studying and in non-study internet use. Further research is necessary to examine if the different sedentary patterns have an effect on the other two health behaviors. Clusters also seemed to be characterized by the parental education level. Adolescents of highly educated parents showed diets of the highest diet quality and lowest levels of sedentarism compared to their peers with lowly educated parents, irrespective of the time spent in MVPA. In previous research, clear associations between socioeconomic factors and dietary habits among adolescents have been found [37,38]. Furthermore, Nilsen et al. [39] revealed that adolescents with higher educated parents, also reported to consume healthier diets. On the other hand, equivocal results have been reported concerning the association between socioeconomic status and the level of MVPA in adolescents [34,40]. Also, the inverse association between parental education and sedentary behavior have been demonstrated in other studies [34]. Although, the Sedentary cluster found in the present study also represented a high amount of adolescents of high educated parents, which leads to the suspicion that the parental education level does not have an influence on the adolescents' sedentary behavior. The difference in defining sedentary behavior, may be an explanation for those contradictory results. While most studies only include TV viewing and PC/video gaming, the present study also included studying and internet use. It is most likely that highly educated parents encourage their children to study more, which in turn can compensate the higher amount of time spent watching TV and/or PC/video gaming, frequently seen in adolescents with lower educated parents. Studies, examining those variables more precisely, are necessary to create a better insight in the determinants of sedentary behavior among adolescents. In the present study, clusters could not be characterized by the FAS. An explanation for not finding any difference, is that FAS is a possible proxy for the education level. For example, the parental education level may be more correlated to eating habits compared to the FAS, which is rather an indicator for someone's affluence rather than someone's knowledge and cognitive performance. Highly educated parents may be more inclined to refer and use information concerning healthy diets compared to low educated parents, irrespective of their income and/or wealth. In the present study, the participation in MVPA was also found to be independent of someone's wealth. These findings are in line with those of Macintyre and Mutrie [41], who established that the total energy expenditure in higher SES youth was not higher compared to lower SES youth. It seemed that adolescents with higher socioeconomic status were more involved in clubs sports, while those with lower SES spent more time in unstructured activities.

A significant association was also found between the clusters and the age category. The present findings indicate that younger adolescents are more physically active and are in line with previous studies showing a decrease in the level of PA with age [11,42]. On the other hand, it appears that younger adolescents eat healthier than older ones [9,43], but this could not be confirmed in the present study. It could be that the age effect disappeared because of the combination with sedentarism within the clusters. TV-viewing has been previously linked to someone's dietary pattern [44] and this is also reflected in the above mentioned clusters, where a high sedentary level is associated with a low DQI-A score and vice versa.

For BMI, no significant differences were found among the five defined clusters, which are in line with the findings of Sabbe et al. [35].

When interpreting the results we have to keep in mind that this study has some limitations. Data for diet quality, MVPA, sedentarism, parent's education and FAS was obtained by self-reporting questionnaires, which has its well known disadvantages (e.g. social desirability, under or over reporting). On the other hand, most of the questionnaires have been tested and validated [16,19,21,22]. Measuring sedentary behaviors has also limitations. Some use screen time, while others include additional sedentary activities (e.g. reading, studying) [4]. It is therefore possible that questionnaires do not reflect the real, total time spent in sedentary behaviors. It should also be stated that this population is an urban population and extrapolation to the general population of European adolescents should be treated cautiously. Furthermore, the low participation rate (59%), due to the inclusion criteria, can be seen as a limitation of the present study.

Strengths of the present study are the large sample size and diverse geographical origin in Europe. The highly standardized procedures used within the HELENA study are also an important strength. Also, this study is one of the first in investigating the clustering of dietary, PA and sedentary behaviors and their socio-demographic factors in this young population. Although, clustering is a relatively new technique in classifying subjects according to several health indices, the defined clusters in the present study, seemed stable and could therefore be seen as representative clusters for European adolescents. It could be interesting for future studies to examine the stability or evaluation of the clusters together with their socio-demographic correlates.

## Conclusion

Firstly, it is important to consider multiple lifestyle related health factors in classifying individuals and when trying to identify high risk groups. Secondly it provides insight in how the health-related behaviors are related in adolescents. It seemed that most adolescents did not present healthy scores on all three included health indices, namely PA, sedentary and dietary behaviors. Differences were found in characteristics of adolescents included in the different clusters. Therefore cluster analyses can be seen as a first step in the development of targeted primary prevention strategies.

## Competing interests

The authors declare that they have no competing interests.

## Authors' contributions

CO participated in the design of the study, data collection, analysis, interpretation of the results and drafted the manuscript. IH was involved in manuscript drafting and coordinated the statistical analysis. IH, IDB, SDH and contributed to the results interpretation, and editing of the manuscript. LAM coordinated the total HELENA study on international level. LAM, JD, FG, MK, YM, DM and KW were involved in the design of the HELENA study and locally coordinated the project. CO, JAT, MMC, MF, JRL and ES performed the data collection locally. All authors participated in the writing of the paper and provided comments on the drafts and approved the final version.

## Pre-publication history

The pre-publication history for this paper can be accessed here:

http://www.biomedcentral.com/1471-2458/11/328/prepub
